# A175 DISEASE ACTIVITY IN FIRST TRIMESTER IS ASSOCIATED WITH REDUCED GROWTH IN INFANTS BORN TO WOMEN WITH INFLAMMATORY BOWEL DISEASE

**DOI:** 10.1093/jcag/gwab049.174

**Published:** 2022-02-21

**Authors:** R Y Wu, P Tandon, L Ambrosio, G Dunsmore, G Wang, N Hotte, L A Dieleman, S Elahi, K Madsen, V Huang

**Affiliations:** 1 University of Toronto Temerty Faculty of Medicine, Toronto, ON, Canada; 2 University of Alberta, Edmonton, AB, Canada

## Abstract

**Background:**

Ulcerative colitis (UC) and Crohn’s disease (CD) are chronic inflammatory bowel diseases (IBD) that affect a significant portion of women in childbearing years. It is known that disease activity in early pregnancy negatively impacts obstetrical and perinatal outcomes, but the impact on infant growth is largely unknown.

**Aims:**

The objective of this study was to compare the growth of infants born to women with active IBD during pregnancy versus those born to women with IBD in remission during pregnancy.

**Methods:**

We conducted a prospective cohort study in a Canadian tertiary centre comprised of 98 pregnant women with IBD (63 with UC and 35 with CD) and 13 healthy pregnant women. We collected maternal demographic at trimester 1 and assessed disease activity at each trimester using clinical disease scores and fecal calprotectin. We then collected perinatal outcomes at delivery and followed the infants’ growth and feeding habits up to 12 months of age.

**Results:**

A total of 103 mother-infant pairs were included in the study, of which 88 infants were born to women with IBD, and 15 born to women with active disease at trimester 1. Active disease at trimester 1 was associated with more adverse obstetrical outcomes, reduced 1-minute and 5-minute APGAR scores and more frequent NICU admissions. Infants born to women with active trimester 1 disease had reduced weight-for-age and length-for-age *Z* scores up to 6 months of age, in the absence of difference in feeding patterns. In addition, women with active disease at trimester 1 had increased expression of IL-8 and IFN-γ compared to those with trimester 1 remission.

**Conclusions:**

Active IBD during first trimester is correlated with decreased infant weight and height up to 6 months of age, suggesting that strict disease control during first trimester, or even preconception, is essential for optimizing infant growth and perinatal outcomes.

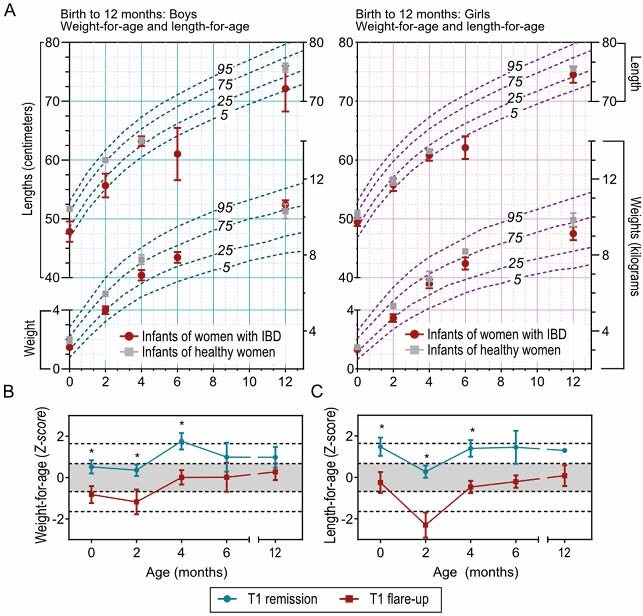

**Funding Agencies:**

None

